# Changes in peripheral immune cells after intraoperative radiation therapy in low-risk breast cancer

**DOI:** 10.1093/jrr/rraa083

**Published:** 2020-10-02

**Authors:** Isabel Linares-Galiana, Miguel Angel Berenguer-Frances, Rut Cañas-Cortés, Monica Pujol-Canadell, Silvia Comas-Antón, Evelyn Martínez, Maria Laplana, Héctor Pérez-Montero, María Jesús Pla-Farnós, Arturo Navarro-Martin, Miriam Nuñez, Brigitte Both, Ferran Guedea

**Affiliations:** Radiation Oncology Department, Hospital Duran i Reynals, Institut Català d'Oncologia (ICO), Avinguda de la Gran Via de l'Hospitalet 199-203, L'Hospitalet de Llobregat, 08098 Barcelona, Spain; Radiobiology and Cancer Group, ONCOBELL Program, Institut d'Investigació Biomèdica de Bellvitge (IDIBELL), Avinguda de la Gran Via de l'Hospitalet 199-203, L'Hospitalet de Llobregat, 08098 Barcelona, Spain; Radiation Oncology Department, Hospital Duran i Reynals, Institut Català d'Oncologia (ICO), Avinguda de la Gran Via de l'Hospitalet 199-203, L'Hospitalet de Llobregat, 08098 Barcelona, Spain; Radiobiology and Cancer Group, ONCOBELL Program, Institut d'Investigació Biomèdica de Bellvitge (IDIBELL), Avinguda de la Gran Via de l'Hospitalet 199-203, L'Hospitalet de Llobregat, 08098 Barcelona, Spain; Radiobiology and Cancer Group, ONCOBELL Program, Institut d'Investigació Biomèdica de Bellvitge (IDIBELL), Avinguda de la Gran Via de l'Hospitalet 199-203, L'Hospitalet de Llobregat, 08098 Barcelona, Spain; Radiobiology and Cancer Group, ONCOBELL Program, Institut d'Investigació Biomèdica de Bellvitge (IDIBELL), Avinguda de la Gran Via de l'Hospitalet 199-203, L'Hospitalet de Llobregat, 08098 Barcelona, Spain; Radiation Oncology Department, Hospital Germans Trias i Pujol, Institut Català d'Oncologia (ICO), Carretera de Canyet, s/n, 08916 Badalona, Spain; Radiation Oncology Department, Hospital Duran i Reynals, Institut Català d'Oncologia (ICO), Avinguda de la Gran Via de l'Hospitalet 199-203, L'Hospitalet de Llobregat, 08098 Barcelona, Spain; Radiation Oncology Department, Hospital Duran i Reynals, Institut Català d'Oncologia (ICO), Avinguda de la Gran Via de l'Hospitalet 199-203, L'Hospitalet de Llobregat, 08098 Barcelona, Spain; Radiation Oncology Department, Hospital Duran i Reynals, Institut Català d'Oncologia (ICO), Avinguda de la Gran Via de l'Hospitalet 199-203, L'Hospitalet de Llobregat, 08098 Barcelona, Spain; Gynecology Department, Hospital Universitari de Bellvitge, Carrer de la Feixa Llarga, s/n, 08907 L'Hospitalet de Llobregat, Barcelona, Spain; Radiation Oncology Department, Hospital Duran i Reynals, Institut Català d'Oncologia (ICO), Avinguda de la Gran Via de l'Hospitalet 199-203, L'Hospitalet de Llobregat, 08098 Barcelona, Spain; Radiobiology and Cancer Group, ONCOBELL Program, Institut d'Investigació Biomèdica de Bellvitge (IDIBELL), Avinguda de la Gran Via de l'Hospitalet 199-203, L'Hospitalet de Llobregat, 08098 Barcelona, Spain; Radiation Oncology Department, Hospital Duran i Reynals, Institut Català d'Oncologia (ICO), Avinguda de la Gran Via de l'Hospitalet 199-203, L'Hospitalet de Llobregat, 08098 Barcelona, Spain; Radiobiology and Cancer Group, ONCOBELL Program, Institut d'Investigació Biomèdica de Bellvitge (IDIBELL), Avinguda de la Gran Via de l'Hospitalet 199-203, L'Hospitalet de Llobregat, 08098 Barcelona, Spain; Medical Affairs & Professional Education, Business Sector Radiotherapy, Medical Technology Business Group, Carl Zeiss Meditec AG, ZEISS Group, Rudolf-Eber-Straße 11 Oberkochen, Germany; Radiation Oncology Department, Hospital Duran i Reynals, Institut Català d'Oncologia (ICO), Avinguda de la Gran Via de l'Hospitalet 199-203, L'Hospitalet de Llobregat, 08098 Barcelona, Spain; Radiobiology and Cancer Group, ONCOBELL Program, Institut d'Investigació Biomèdica de Bellvitge (IDIBELL), Avinguda de la Gran Via de l'Hospitalet 199-203, L'Hospitalet de Llobregat, 08098 Barcelona, Spain

**Keywords:** immune system, immunomodulation, flow cytometry, immunophenotyping, breast cancer, intraoperative radiation therapy

## Abstract

A detailed understanding of the interactions and the best dose-fractionation scheme of radiation to maximize antitumor immunity have not been fully established. In this study, the effect on the host immune system of a single dose of 20 Gy through intraoperative radiation therapy (IORT) on the surgical bed in low-risk breast cancer patients undergoing conserving breast cancer has been assessed. Peripheral blood samples from 13 patients were collected preoperatively and at 48 h and 3 and 10 weeks after the administration of radiation. We performed a flow cytometry analysis for lymphocyte subpopulations, natural killer cells (NK), regulatory T cells (Treg) and myeloid-derived suppressor cells (MDSCs). We observed that the subpopulation of NK CD56^+high^ CD16^+^ increased significantly at 3 weeks after IORT (0.30–0.42%, *P* < 0.001), while no changes were found in immunosuppressive profile, CD4^+^CD25^+^Foxp3^+^Helios^+^ Treg cells, granulocytic MDSCs (G-MDSCs) and monocytic MDSCs (Mo-MDSCs). A single dose of IORT may be an effective approach to improve antitumor immunity based on the increase in NK cells and the non-stimulation of immunosuppressive cells involved in immune escape. These findings support future combinations of IORT with immunotherapy, if they are confirmed in a large cohort of breast cancer patients.

## INTRODUCTION

Breast cancer is the most common malignancy and the second highest cause of death in women worldwide [1]. The molecular mechanisms of breast cancer, particularly tumor cell immune evasion from the host immune response, have been the focus of intense research. The immune system appears to play an essential role in the initiation of breast cancer, progression and response to treatment, given that immune activation is associated with a higher pathological response [2]. The current treatment for early-stage breast cancer is breast conservation surgery followed by radiation therapy. The tumor bed is a preferential site of relapse. Increased local control is associated with improved overall survival, so events close to the tumor bed are crucial for the final prognosis [3]. Several trials have evaluated various techniques of accelerated partial breast irradiation (APBI) of the lumpectomy bed vs conventional whole-breast irradiation in terms of efficacy and other outcomes [4, 5]. The results indicate that treatment with intraoperative radiation therapy (IORT) yields similar results in terms of survival and toxicity as whole-breast radiation therapy, with the benefit of reduced treatment time. In this context, IORT has been established as a good option for patients’ treatment in early-stage breast cancer undergoing conserving surgery, or in combination with external beam radiation therapy (EBRT) in patients with risk factors [4, 6].

Research on the interactions between radiation therapy and the immune system has shown new mechanisms that can be exploited to improve the efficacy of radiotherapy [7, 8]. Radiobiology has focused on normo-fractionated schemes, but little is known about the biological effects of high doses per fraction. Radiotherapy can induce the release of antigens during the death of cancer cells that, in association with proinflammatory signals, activate the innate immune system by inducing the activation of specific T cells against the tumor. Radiotherapy also has effects on the tumor microenvironment, favoring the infiltration of activated T lymphocytes and overcoming some of the barriers of antitumor rejection [9, 10]. However, radiotherapy exerts immune inhibitory effects too. The limited availability of intratumoral dendritic cells to present radiation-released antigens and the increase in the irradiated tumor microenvironment of differents factors, including, e.g. transforming growth factor (TGF) β and immnusopressive cells, such as myeloid-derived suppressor cells (MDSC) and regulatory T (Treg) cells, are part of theses immune inhibitory effects [11]. Studies have shown that conventional fractional EBRT produces an increase in both the levels of MDSCs and Treg cells characterized by systematically expanding and promoting T cell dysfunction [12]. However, it has been reported that the highest single doses applied in hypo-fractionated schemes are more advantageous for stimulating the immune system [7, 9]. Hypo-fractionated radiotherapy inhibits hypoxia and reduces the recruitment of immunosuppressive cells into primary tumors, generating a microenvironment with lower Programmed Death-Ligand (PD-L1), releasing the inhibition on cytotoxic CD8+ and boosting not only the local but also systemic anti-cancer immunity [13]. This approach has been explored in radiotherapy techniques using high-dose fractionations such as stereotactic body radiotherapy (SBRT) [14].

IORT involves precise delivery of a large dose of ionizing radiation to the tumor bed, which is the area of higher risk of relapse. Although conclusive data on immune reactions and IORT are not available, it is likely that the single high doses administered during IORT cause immunogenic cell death of cancer cells remaining *in situ* after surgery by activation of the immune system [15]. There are some published investigations on the effect of IORT on tumor cells as well as wound fluid [16–18]. The results suggest that higher radiation doses result in increased tumor cell necrosis and antigen presentation, and recruitment of T-cells to irradiated and possibly distant unirradiated tumors, providing an additional reason to investigate IORT anew, perhaps in combination with immune checkpoint blockade treatments [19, 20]. Therefore, systemic functional changes in response to high doses of radiotherapy delivered in the tumor bed by IORT as a single treatment modality were investigated in this preliminary study. We assessed changes in peripheral blood immune cell composition after IORT on the surgical bed in patients with low-risk breast cancer. The information provided by this study could be useful to suggest new combinations and timings of IORT and immunotherapy in breast cancer patients.

## MATERIALS AND METHODS

The pilot study protocol was approved by the Clinical Research Ethical Committee of the hospital (protocol code IORT-01, reference PR009/16), and written informed consent was obtained from all participants. From October 2016 to September 2018, a total of 13 patients diagnosed with low-risk breast cancer (T1N0M0 and T2N0M0) who were candidates for breast-conserving surgery and IORT were eligible to participate in the study. Inclusion criteria were as follows: age >50 years, histologically proven invasive ductal carcinoma <3.5 cm, low-risk luminal A subtype (estrogen-receptor and/or progesterone-receptor positive), HER2 negative, and protein Ki-67 <20%, without lymph node involvement (N0) adequate for breast-conserving surgery and IORT (lesion distance to skin or breast wall ≥0.7 cm), as well as capacity to understand the characteristics of the study and to give informed consent. Exclusion criteria were the presence of bilateral breast cancer at the time of diagnosis, a previous history of malignant disease except for non-melanoma skin cancer, need for treatment with chemotherapy, and state of immunosuppression, both current and developed during follow-up, caused by immunosuppressant medications or systemic disorders. For each patient, the postoperative pathological report was reviewed, and when high-risk factors were found, such as grade 3 tumor differentiation, lymphovascular invasion, triple-negative breast cancer subtype, positive nodes and affected margins or close margins <5 mm without re-excision, these cases were excluded from the analysis. Patients with these characteristics underwent EBRT with or without prior chemotherapy.

Intraoperative radiotherapy was administered using the INTRABEAM system (Carl Zeiss Surgical, Oberkochen, Germany) emitting low-energy (50 kV) photons at a high dose-rate to the target tumor bed volume, with rapid dose fall-off and hence limited exposure to adjacent non-tumor tissues [21]. Spherical applicators were used to deliver a uniform dose at the inner-surface of the breast lumpectomy cavity. The size of the applicator used in each case was based on the largest one that fits comfortably to the tumor bed so that the skin and subcutaneous tissue can be fixed with a suture on the bag over the sphere. The applicator was kept at least 0.7 cm from the skin. The dose rate varied according to the diameter of the applicator and the energy of the beam. A single dose of 20 Gy (in water) was prescribed and delivered in a period of 15–35 min, depending on the size of the applicator. IORT was administered immediately after removal of tumor volume when R0 resection was obtained.

### Flow cytometry

Peripheral blood samples (9 mL) drawn in ethylenediaminetetraacetic acid (EDTA) were obtained preoperatively before surgery and IORT, and at 48 h and 3 and 10 weeks after IORT. Fresh blood was used, and the samples were processed within 24 h.

Blood (50–100 μL) of blood was added to the appropriate tubs, and cells were processed according to the manufacturer’s instructions. The antibody panels used were the following: lymphocyte phenotyping panel (DuraCloneTM, Beckman Coulter Life Sciences, Indianapolis, IN, USA): CD16, CD56, CD19, CD14, CD4, CD8, CD3 and CD45 antibodies; regulatory T cells panel (DuraCloneTM, Beckman Coulter Life Sciences): CD45RA, CD25, CD39, CD3, CD45, CD4, Helios and intracellular Foxp3 antibodies; and myeloid-derived suppressor cells panel (DuraCloneTM, Beckman Coulter Life Sciences): CD45, HLA-DR, CD14, CD33 and CD11b antibodies.

All flow cytometry data were acquired on a 10-color/3-laser Gallios flow cytometer (Beckman Coulter). The instrument has not been altered. The stability of the flow cytometer was assured through a quality control procedure using Flow-Check Pro Fluorospheres (Beckman Coulter). A compensation matrix for each panel was created using the compensation tubes supplied with each panel, according to manufacturer’s instructions. A minimum number of 100 000 leucocytes for lymphocyte phenotyping, 100 000 leucocytes for MDSC analyses and 40 000 leucocytes for regulatory T cell analysis was established [22, 23]. Data were manually analyzed using FlowJo software v. 10.5 (Tree Star Inc., Ashland, OR, USA).

### Statistical analysis

Categorical data are expressed as frequencies and percentages, and continuous data as median and range. Friedman one-way Analysis of Variance (ANOVA) was used to compare changes of cell populations at different time periods and the Wilcoxon signed-rank test to assess differences in the cell populations at different time periods as compared to each baseline. Statistical significance has been confirmed by Conover *post hoc* test.

Statistical significance was set at *P* < 0.05. Data were analyzed using the R statistical program (version 3.5.0).

## RESULTS

A total of 13 women diagnosed with early-stage breast cancer were included in the study. The salient characteristics of these patients are shown in Table 1. The median age was 66 years (range 61–85 years). Most lesions were located in the upper outer quadrant (69.2%) of the left breast (84.6%). The most common form was the T1 stage (92.3%). In all patients, the histological diagnosis was infiltrating ductal carcinoma, luminal subtype. All patients received hormonal therapy. The most common size of the applicator was 3.5 cm (61.5% of the cases), with a mean treatment time of 19 min. No relapse was observed, and after a median follow-up time of 14 months, 100% of patients remained alive without disease.

**Table 1:** Characteristics of 13 patients with low-risk breast cancer; data are presented as frequencies with percentages in parenthesis unless otherwise stated

	*n* (%)
Age, years, median (range)	66 (61–85)
Stage	
T1b	6 (46.1)
T1c	6 (46.1)
T2	1 (7.7)
Tumor grade	
I	7 (53.8)
II	6 (46.2)
Hormone receptor status	
Estrogen receptor positive	13 (100)
Progesterone receptor positive	10 (76.9)
Progesterone receptor negative	3 (23.1)
HER2 negative	13 (100)
Ki67	
<14%	10 (76.9)
>14%	3 (23.1)
Associated carcinoma *in situ*	6 (46.1)
Applicator size, cm	
3	2 (15.4)
3.5	8 (61.5)
4	3 (23.1)
Treatment time, min, median (range)	19 (18–28)
Hormone therapy	13 (100)
Tamoxifen	8 (61.5)
Letrozole	5 (38.5)

### Immunophenotyping panel

**Fig. 1.**
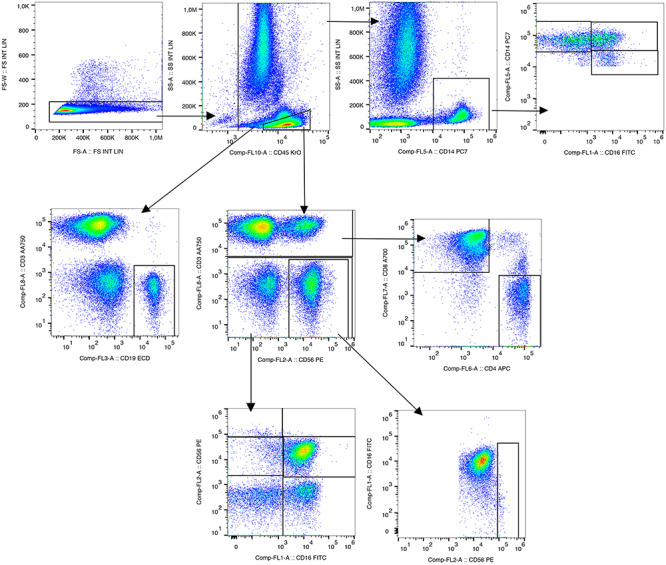
Immunophenotyping panel for the detection of different populations and subpopulations of lymphocytes and NK cells.

Of the 52 samples obtained at different time intervals in the course of IORT, 44 were analyzed. The flow cytometry gating strategy used for the analysis of all cell types is shown in Fig. 1. Lymphocytes were identified from CD45. T lymphocytes were distinguished from total lymphocytes using the characteristic CD3 marker. From these, CD4^+^ helper lymphocytes were separated from CD8^+^ cytotoxic. B lymphocytes were identified by characterization CD3^−^ CD19^+^. To select the NK cells, a negative selection of the CD3^−^ CD19 ^−^CD20^−^ markers was made; subsequently through the combination of CD56 and CD16, subpopulations of NK cells were obtained.

**Fig. 2.**
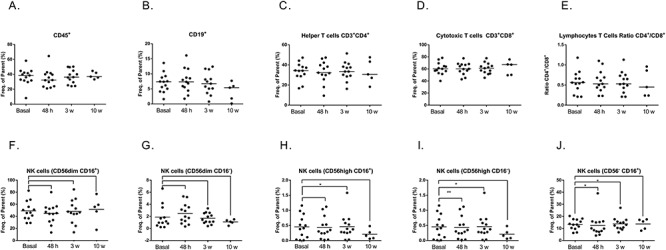
Median values of CD45^+^ cell population (**A**), CD19^+^ (**B**), helper T cells CD3^+^ and CD4^+^ (**C**), cytotoxic T cells CD3+ CD8+ (**D**), lymphocytes T cells ratio (CD4^+^/CD8^+^) (**E**), NK cells (CD56dim CD16^+^) (**F**), NK cells (CD56dim CD16^−^) (**G**), NK cells (CD56high CD16^+^) (**H**), NK cells (CD56high CD16^−^) (**I**) and NK cells (CD56^−^CD16^+^) (**J**). ^*^P < 0.05 when Wilcoxon’s signed-rank test was applied.

Changes of total lymphocytes (CD45^+^) along the study period after the administration of IORT were not statistically significant (Friedman one-way ANOVA, *P* = 0.356). Also, differences in the paired comparisons (different time points vs baseline) were not statistically significant (Wilcoxon signed-rank test, *P* = 0.5, 0.95, 0.81 at 48 h, 3 and 10 weeks, respectively) (Fig. 2A). When the different lymphocyte cell subpopulations were assessed, a progressive decrease in CD19^+^ B cells was observed from 48 h after IORT, reaching minimal values at 10 weeks (Friedman one-way ANOVA, *P* = 0.007). Although no statistically significant differences were found when the percentage of B cells in the baseline (7.38%) was compared with that found 10 weeks after IORT (5.91%), a trend towards significance was found (Wilcoxon signed-rank test, *P* = 0.062) (Fig. 2B). The subpopulation of T helper cells CD3^+^ CD4^+^ did not reveal any significant statistical differences among the different comparisons performed, although a slight decrease after IORT as compared to baseline (34.20%), reaching minimal values 10 weeks after irradiation (30.60%), was observed (Fig. 2C). By contrast, changes in CD3^+^ CD8^+^ cytotoxic T cells showed a progressive increase during the study period, with the highest values at 10 weeks as compared with baseline (67.10 vs 60.20%), but these differences did not reach statistical significance either when the changes of cell populations at different time periods or when the different time periods were compared with the baseline (Fig. 2D). The CD4^+^/CD8^+^ ratio showed an increase in cytotoxic lymphocytes from 0.56 to 0.45% at 10 weeks, but this change was not statistically significant (Wilcoxon signed-rank test, *P* = 0.58) (Fig. 2E).

**Fig. 3.**
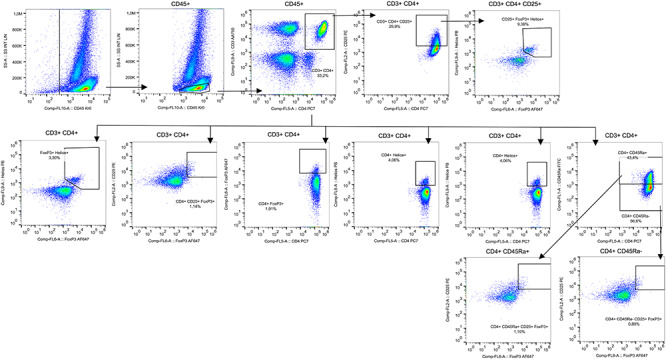
Immunophenotyping panel for regulatory T cells.

In the subpopulation of NK cells defined as CD56^dim^ CD16^+^, there was a decrease from baseline to 48 h, but the difference was not significant. Differences between 3 and 10 weeks were not significant, although we observed a slight increase when compared with the baseline (Fig. 2F). In the population of NK cells with activated phenotype defined as CD56^+high^ CD16^+^, differences along the study period after the administration of IORT were significant (Friedman one-way ANOVA, *P* = 0.007). When the different time periods were compared with the baseline there was a significant increase at 3 weeks compared with the baseline (0.30–0.42%, Wilcoxon signed-rank test, *P* = 0.001) (Fig. 2H). On the other hand, in the subpopulations of CD56^dim^ CD16^−^ and CD56^+high^ CD16^−^, changes were not statistically significant in either of the comparisons by Friedman’s test or the Wilcoxon’s test for paired comparisons at different time points vs baseline (Fig. 2G and I). In the subpopulation of CD56^−^CD16^+^, there was an increase from 13.20% at baseline to 15.80% at 10 weeks, but these differences were not statistically significant using Wilcoxon’s test (*P* = 0.3125) nor for the comparisons at 48 h and 3 weeks. Also, differences were not significant when changes of cell populations at different time periods were compared (Friedman’s test, *P* = 0.05) (Fig. 2J).

**Fig. 4.**
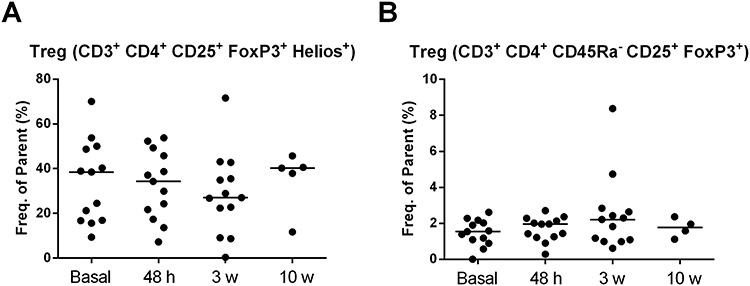
Median values of the CD4^+^ CD25^+^ Foxp3^+^ Helios^+^ cell population (**A**) and CD4^+^ CD25^+^ Foxp3^+^ CD45RA^−^ (**B**) during the study period at different time points.

### Treg cells

The strategy used for the analysis of different phenotypic and functional Treg subsets was screening lymphocytes according to side scattering and forward scattering characteristics, which were additionally blocked for CD4^+^ T cells. Then, CD4^+^ T cells were examined for CD4^+^ CD25^+^ Foxp3^+^ Helios^+^ cell populations. Treg cells were also divided into functional subsets based on CD45RA and Foxp3 expression. The representative diagrams of flow cytometry for the analysis of all cell types are shown in Fig. 3.

When changes in the cell populations at different timepoints were compared with the baselines, a slightly decreased CD3^+^ CD4^+^ CD25^+^ Foxp3^+^ Helios^+^ cell population was observed at 48 h after the administration of IORT, with the lowest values at 3 weeks (27 vs 38.50% at baseline), although no significant differences were observed in any of the comparisons. Changes in the CD3^+^ CD4^+^ CD45RA^−^ CD25^+^ Foxp3^+^ subset at different timepoints were not observed (Friedman’s test, *P* = 0.277) (Fig. 4A and B).

### MDSCs

**Fig. 5.**
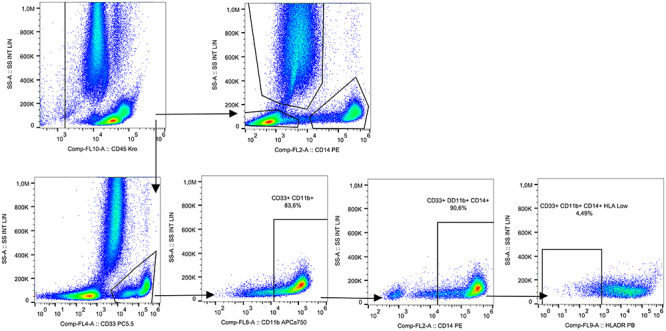
Immunophenotyping panel for MDSCs.

Representative diagrams of flow cytometry for the analysis of all cell types are shown in Fig. 5. MDSCs were defined as CD45 ^+^ CD33 ^+^ CD11b ^+^ cells. Then, MDSC cells were also divided into subsets based on CD14 and HLA-DR expression.

**Fig. 6.**
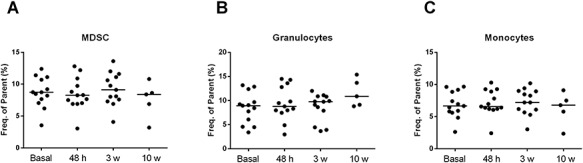
Median values of MDSCs (**A**), G-MDSCs (**B**), and Mo-MDSCs (**C**) during the study period at different time points.

Statistically significant changes in the overall MDSC population were not observed (Fig. 6A). For granulocytic MDSCs (G-MDSC) characterized as CD33^+^ CD11b^+^ CD14^−^ and monocytic MDSCs (Mo-MDSC) characterized as CD33^+^ CD11b^+^ CD14^+^ HLADR^−/low^, statistically significant differences were not found. Both in the G-MDSC and Mo-MDSC subsets, median percentages at different time points as compared with baseline were not statistically significant. Similar results were obtained when changes along the study period after the administration of IORT were assessed with Friedman’s test (G-MDSC, *P* = 0.471; Mo-MDSC, *P* = 0.782) (Fig. 6B and C).

## DISCUSSION

Results presented in this study indicate that treatment with IORT in low-risk breast cancer patients undergoing conserving breast surgery can affect the balance of immune cells in the peripheral blood. During the study period, we noted that radiation therapy increased the presence of peripheral NK cells while no changes were detected in the immunosuppressive profile, characterized by Treg cells and MDSCs.

Radiation therapy can cause apoptosis of T cells and lymphopenia [24]. In the present study, statistically significant differences in the total number of peripheral blood lymphocytes were not found, but the population of B lymphocytes experienced a decrease after IORT, reaching its lowest values at 10 weeks. For the different subpopulations of T lymphocytes, a progressive increase in cytotoxic T lymphocytes throughout the study period was observed. The CD4^+^/CD8^+^ ratio showed an increase of cytotoxic lymphocytes at 10 weeks as compared to baseline values. Differences, however, did not reach statistical significance probably due to the limited number of patients included in the study. Recent evidence suggests that T cells have an important function in regulating cancer growth. Several T cells play a critical role in the balance of anti- and pro-tumor immune responses within the tumor microenvironment as well as in the systemic circulation [25, 26]. In fact, cytotoxic T cells are the main final effectors of tumor cell death. However, Treg cells generally suppress or downregulate the induction and proliferation of cytotoxic cells. Therefore, the dominance of cytotoxic T lymphocytes is a favorable prognostic factor in cancer [27, 28].

We found significant changes in the subpopulation of NK cells in breast cancer patients treated with IORT. There is evidence that irradiation-induced tissue injury increases the expression of NK-activating ligands (e.g. NKG2D ligands) on malignant cells, thereby rendering tumors more susceptible to NK cell cytotoxic activity, modulating the immune response by increasing antigenicity and upregulating the expression of proinflammatory cytokines and chemokines [28, 29]. CD56^dim^ NK cells have a high cytotoxic activity and express high levels of perforin; in addition, the high expression CD16 makes them efficient mediators of antibody-dependent cellular cytotoxicity. CD56^+^ CD16^−^ NK cells have a lower cytotoxic capacity but are the most efficient cytokine producers, including interferon-γ (IFN-γ), tumor necrosis factor-α (TNF-α), Interleukin-12 (IL-12), IL-15 and granulocyte and monocyte colony-stimulating factor [30]. In the present study, the subset CD56^dim^ CD16^+^ NK cells showed an increase after 3 weeks of IORT and continued to increase until the end of the follow-up period (46.20–55.80%). The CD56^−^ subset expressing CD16 at high density, but showing functional alterations due to its low cytotoxic activity and cytokine production [28], presented variations during the study period. Although there was a decrease at 48 h, the percentage of cells increased at 3 and 10 weeks. We observed that the only significant increase was in the subset CD56^high^ CD16^+^ NK cells, especially at 3 weeks (0.30–0.42%). CD56^high^CD16^+^ NK cells are characterized by NKG2A and low levels of perforin, and are primarily specialized for cytokine production. In peripheral blood these NK cells express CD62L, CCR7, CXCR4 and CXCR3 that allows their preferential recruitment to secondary lymphoid organs, tumors and inflamed tissues [31]. One of the factors responsible for the variations in the percentage of these cells during our study period could be the presence of cytokine release, especially the overexpressed TGF-β molecule after irradiation. *In vivo* studies in breast cancer patients have shown a positive correlation of TGF- β1 with NKG2A receptor expression, thus suggesting that this molecule plays a role in these regulatory mechanisms [32]. These findings indicate that a single radiation dose might increase the number of these cells that have antitumor capacity. The identification of NK cell subsets endowed with particular functional capabilities might help monitor residual antitumor NK cell-mediated responses in breast cancer patients.

Treg cells can promote tumor progression by inhibiting effective antitumor immunity. These cells are more resistant to ionizing radiation than conventional CD4^+^ T cells and show a relative increase after radiotherapy [33–35]. In our study, the percentage of peripheral blood CD4^+^ CD25^+^ Foxp3^+^ Helios^+^ Treg cells did not experience significant change during the study period. This relevant finding indicates that the use of this single high-dose approach may be essential to neutralize these immunosuppressive cells, which are one of the main components of immune escape. In recent years, there has been great interest in promoting the production of more efficient Treg cells enhancing antitumor activity [36]. In this respect, assessment of Treg cell levels and related cytokines in the peripheral blood may be important to guide individualized treatments in breast cancer.

It has been shown that the prevalence of MDSCs with immunosuppressive activities is an important mechanism of tumor escape. In mouse models, accumulated evidence has demonstrated the importance of MDSCs in the development and progression of breast cancer [37]. Gonda *et al*. [38] examined MDSC levels in 155 patients with breast cancer. MDSCs in circulating peripheral blood increased in patients with breast cancer compared to healthy volunteers. Also, the levels of MDSCs in preoperative patients and in patients with recurrent breast cancer were significantly higher compared with postoperative patients, patients with recurrent breast cancer who received chemotherapy and healthy volunteers. Studies have shown that RT, especially conventional fractional RT, produces an increase in the levels of MDSCs characterized by being more resistant to radiation [9, 39]. However, in our study, changes in the overall MDSCs population as well as in G-MDSC and Mo-MDSC subpopulations were not observed. This finding is in line with the results previously published by our group in patients with lung cancer. We also observed that the use of high doses in hypofractionated schemes, in this case stereotactic body radiotherapy (SBRT), did not produce an increase in the percentage of MDSCs when analyzed in peripheral blood. Indeed a significant decrease in the percentage of granulocytes was observed 6 months after the administration of radiation. These results may show the possible benefit of the application of this type of scheme, which seems to be more effective in controlling cells related to immunosuppression [40].

In this respect, studies have been developed to test if hypofractionated dose schedules may be more effective than conventional fractionated radiotherapy. In an experimental study of mice bearing murine melanoma treated with up to 15 Gy radiation given in various-size fractions, Schaue *et al*. [41] found that fractionated treatment with medium-size radiation doses of 7.5 Gy/fraction gave the best tumor control and tumor immunity while maintaining low Treg cells number. In a preclinical study, Lee *et al*. [42] compared a single 20 Gy dose with 5Gy x 4 over 2 weeks. Ablative 20 Gy radiation dramatically increased T-cell priming in draining lymphoid tissues, leading to reduction/eradication of the primary tumor or distant metastasis in a CD8^+^ T cell-dependent fashion, whereas fractionated radiation therapy showed a lower inhibition of tumor growth. However, the best dose-fractionation regimen to maximize antitumor immunity remains to be established.

In this preliminary study, we show that the administration of a single dose of IORT altered the balance of peripheral immune cells by increasing NK cells but did not produce changes in Treg cells or MDSCs. No previous studies have evaluated the effect of IORT on peripheral immune cells in breast cancer patients. We therefore consider that these results help to clarify the interactions of IORT with the host immune system and add evidence to suggest IORT utilization as an attractive alternative to whole-breast irradiation in selected patients with early-stage breast cancer. However, given the exploratory nature of our study, more studies are needed, with a greater number of patients and a control group of patients receiving hypofractionated whole-breast irradiation, to support the use of the combination of IORT with immunotherapy as a new therapeutic option in breast cancer patients.

## Supplementary Material

supplementary_material_rraa083
